# Using EMS data to explore community-level factors associated with firearm violence in North Carolina

**DOI:** 10.1186/s40621-024-00539-9

**Published:** 2024-10-25

**Authors:** Yuni Tang, Nandi L. Taylor, Lucas M. Neuroth, Kathleen A. Higgins, Anna E. Waller, Stephen W. Marshall, Katherine J. Harmon

**Affiliations:** 1 Highway Safety Research Center, University of North Carolina at Chapel Hill, Chapel Hill, USA; 2 Injury Prevention Research Center, University of North Carolina at Chapel Hill, Chapel Hill, USA; 3 Department of Emergency Medicine, University of North Carolina at Chapel Hill, Chapel Hill, USA; 4 Department of Epidemiology, University of North Carolina at Chapel Hill, Chapel Hill, USA

## Abstract

**Background:**

Firearm violence is a significant public health issue. However, it is unclear if there is an association between the Social Vulnerability Index (SVI) and the intent of both fatal and nonfatal firearm injuries, and if these associations are modified by community race and ethnic composition. This study examines the association between community-level social vulnerability and firearm injury incidence in North Carolina (NC) using 2021–2022 emergency medical services (EMS) data. Additionally, it investigates how these associations vary by the intent of injury (assault, self-inflicted, and unintentional), and whether they are modified by community racial/ethnic composition.

**Methods:**

This cross-sectional study utilized NC EMS data, capturing firearm incidents from January 1, 2021, to December 31, 2022. The SVI from the Centers for Disease Control and Prevention (CDC) was used to assess community-level vulnerability. The SVI’s racial/ethnic minority status component was removed for stratification analysis. Firearm injury rates were calculated per 100,000 population, and negative binomial regression models were used to estimate Incidence Rate Ratios (IRRs) for different SVI levels and intents of firearm injuries.

**Results:**

During the study period, we identified 7,250 EMS encounters at non-healthcare locations related to firearm injuries, encompassing 2,648 NC census tracts. Assault was the leading cause of firearm injuries (*n* = 3,799), followed by self-inflicted (*n* = 1,498), and unintentional injuries (*n* = 722). High-SVI communities had significantly higher rates of firearm injuries compared to low-SVI communities, particularly for assault-related injuries. When the minority status component was excluded from SVI, racial/ethnic minority status emerged as a significant modifier, with higher rates of firearm injuries being observed in communities with larger racial/ethnic minority populations.

**Conclusion:**

Community-level social vulnerability is significantly associated with firearm injury incidence, with the effect being more pronounced in racial/ethnic minority communities. These findings underscore the need for targeted public health interventions that address underlying social determinants of health (e.g., access to education) to reduce firearm violence. Future research should further explore the intersection of social vulnerability and racial/ethnic composition to develop effective prevention strategies.

**Supplementary Information:**

The online version contains supplementary material available at 10.1186/s40621-024-00539-9.

## Introduction

The nature and frequency of firearm violence, together with its impact on Americans’ health and safety, make it a major public health concern. Firearm injuries are among the top five causes of death for people aged 1–44 years in the United States (US), representing 7.9% of premature death or years of potential life lost before the age of 65 (Centers for Disease Control and Prevention 2024). In the US, there were 42,967 firearm fatalities in 2023, with deaths increasing by 43% between 2010 and 2020. Suicide by firearm accounted for most of these fatalities (56%), with homicide and unintentional firearm deaths accounting for 35% and 4% of firearm fatalities, respectively (The National Institute for Health Care Management Foundation 2024). For each firearm fatality, more than two persons are nonfatally injured, with most nonfatal injuries being assaults (Schildkraut and Carter 2022). These statistics underscore the urgent need for comprehensive interventions to prevent and mitigate the devastating consequences of firearm violence on communities across the country.

Firearm violence impacts demographic groups disproportionately. Young people (20–39 years of age), males, and Black individuals have the highest firearm injury rates and experienced the largest rate increases in recent years (Kegler et al. 2022; Rees et al. 2022). Additionally, previous studies have highlighted the value of using healthcare data, such as Emergency Medical Services (EMS) (Rowh et al. 2024)and Emergency Department (ED) (Van Dyke et al. 2022; Senior et al. 2023)data, to better understand firearm injuries, capture firearm injury rates, identify the risk factors on both individual and community levels, and further inform violence prevention and intervention strategies, including community violence prevention programs (Hink and Hink 2021; Kwon et al. 2023). Additionally, while individual-level risk factors are important, recent studies have demonstrated that risk patterns extend beyond the individual to the community level. For example, several studies have demonstrated that firearm fatality rates are higher in communities with higher social vulnerability, as measured by the Social Vulnerability Index (SVI) (Van Dyke et al. 2022; Polcari et al. 2023; Dirago et al. 2024). SVI is a publicly available, validated measure of community vulnerability calculated nationally at the census-tract and county level, that can provide some context to underlying conditions that contribute to community-level firearm related injury in the US (Agency for Toxic Substances and Disease Registry 2020). Furthermore, several studies have examined the association between SVI and community-level firearm related injuries using healthcare datasets (e.g., EMS) which may provide further context beyond police-reported firearm injuries (Van Dyke et al. 2022;  Wulz et al. 2023; Spitzer et al. 2022).

SVI is a composite score composed of many demographic and economic community characteristics (e.g., access to education, housing, and transportation), that align closely to social determinants of health, many of which are associated with sustained negative public health outcomes. Since its inception in 2011, SVI has incorporated community-level racial and ethnic composition into its composite score. However, its inclusion can conflate identifying minoritized populations as an indicator of vulnerability while ignoring longstanding impacts of historical social and structural factors on these populations. In addition, aggregating these community-level factors precludes the stratification of results by community racial and ethnic composition, potentially masking aspects of the complex interplay between race, measures of socioeconomic vulnerability, and firearm violence at the community-level. The 2020 edition of SVI allows for the disaggregation of minority status, enabling the examination of race/ethnicity as an effect measure modifier in the relationship between SVI and firearm violence.

To date, most studies have focused on firearm-involved assaults, so it is unclear if there is an association between SVI and intentionally self-inflicted or unintentional firearm injuries, and if these associations are modified by community race and ethnic composition. Furthermore, this is the first application of EMS data to investigate the association between SVI and non-fatal firearm injury in North Carolina (NC). We aim to address these gaps by examining community-level social vulnerability and rates of firearm violence in NC using 2021–2022 EMS data. The study further aims to investigate associations between firearm injury incidence and SVI by intent and community racial and ethnic composition.

## Methods

### Data source

Firearm incident data were obtained from the NC Office of EMS (OEMS) for the period of January 1, 2021, through December 31, 2022. These data are collected as part of the North Carolina Disease Event Tracking and Epidemiologic Collection Tool (NC DETECT), NC’s statewide public health syndromic surveillance system. We additionally examined measures of social vulnerability, as well as community characteristics, using the Centers for Disease Control and Prevention (CDC)/ Agency for Toxic Substances and Disease Registry’s (ATSDR) 2020 SVI dataset (Centers for Disease Control and Prevention, CDC 2020). The census-tract population estimates were obtained from 2016 to 2020 American Community Survey (ACS) data.

## Measurements

### Identifying firearm injury incidents

The study population was limited to the NC EMS encounters for the treatment of incident injuries related to the discharge of a firearm at non-healthcare locations. Initial firearm injury encounters were identified by firearm-related ICD-10-CM codes in the Injury Cause field, a mention of gunshot wound in the chief complaint, or a dispatch complaint of “Stab/Gunshot Wound/Penetrating Trauma” and mention of gunshot wound in the EMS narrative (Snyder et al. 2024). Abbreviations, synonyms, and misspellings were included in the definition to maximize sensitivity. Each EMS encounter was manually reviewed for the removal of false positives and to manually assign intent (intentional-assault, intentional-self-inflicted, unintentional, or undetermined). False positives included subsequent encounters for a previous firearm injury; near misses; injuries resulting from bb guns, air guns, pellet guns; and injuries resulting from firearms used as a blunt weapon, e.g., pistol whips (Snyder et al. 2024).

### Geocoding process

Once processed, the EMS data were geocoded and linked with geospatial data using ArcGIS (ESRI; Redlands, CA). The pre-installed ESRI Address Locator was used to geocode firearm incident location (“Incident Address”). Unmatched and tied addresses were manually reviewed and geocoded. Incidents with missing location information that could not be geocoded were excluded from analysis. We excluded 2% of records due to missing information (*n* = 176), which included 76 unmatched locations and 100 tied locations. The final dataset of geocoded records contained 7,250 locations tied to incidents of firearm violence.

### SVI

The CDC/ATSDR’s SVI is a tool used to identify communities at heightened risk of substantial human and economic losses following disasters caused by natural or human factors (Kim et al. 2019). It has been employed to assess the impact of population-level social characteristics on chronic diseases and injuries, including firearm injuries (Polcari et al. 2023;  Neelon et al. 2021; Jain et al. 2022). The SVI incorporates 16 social variables organized into four categories: Socioeconomic status (Theme 1), household composition and disability (Theme 2), racial and ethnic minority status (Theme 3), and housing type and transportation (Theme 4). Drawing on data from the American Community Survey 2016–2020 5-year estimates, each census tract in the US is assigned a ‘score’ ranging from 0 (indicating lower vulnerability) to 1 (indicating higher vulnerability) (Hink and Hink 2021).

### Community racial and ethnic composition

The recently updated 2020 edition of SVI allows for the disaggregation of components from the composite variable, including the disaggregation of minority status, enabling the examination of race/ethnicity as a potential modifier in the relationship between SVI and firearm violence. Therefore, we removed Theme 3 (racial and ethnic minority status) from the overall SVI, and a new overall SVI was recalculated. For our analysis, we defined a racial and ethnic minority population as one including Hispanic or Latino (of any race), Non-Hispanic (NH) Black, NH American Indian and Alaska Native, NH Asian, NH Native Hawaiian and Other Pacific Islander, NH two or more races, NH other races. These racial categorizations are based on the US Census ACS data (U.S. Census Bureau 2022). To examine community racial and ethnic composition as a possible modifier, we further stratified by census tracts with a racial/ethnic minority composition above the 50th percentile, which is classified as having high racial/ethnic minority population (“high”), versus communities at or below its percentile (“low”).

### Statistical analysis

Firearm injuries and SVI data were merged by census tract. Twelve census tracts with a population of zero were removed from the analysis, which contained two cases of assaults and one case for which injury intent was undetermined. Additionally, undetermined firearm injuries were not included in the analysis. Descriptive statistics were used to summarize characteristics of communities (as indicated by census tract) with firearm injuries treated by NC EMS between 2021 and 2022. Means, medians, standard deviations (SD), and interquartile ranges (IQR) were calculated, with results stratified according to firearm injury intent (Additional file 1:Table S1). In addition to summary statistics, firearm injury incidence rates were calculated for each census tract per 100,000 using the 2020 US Census data as the population denominator. To examine the association between firearm injury incidence and levels of social vulnerability, we used a negative binomial model to account for overdispersion. to estimate Incidence Rate Ratios (IRRs), with two-sided p-values and a significance level of 0.05. We modeled the relationship between social vulnerability and firearm injury incidence, overall and by firearm injury intent. We elected to plot IRRs using a natural (i.e. non-logarithm) y-axis, since all IRRs were above the null and had approximately similar precision. Statistical analyses were conducted using SAS version 9.4.

## Results

**Fig. 1:**
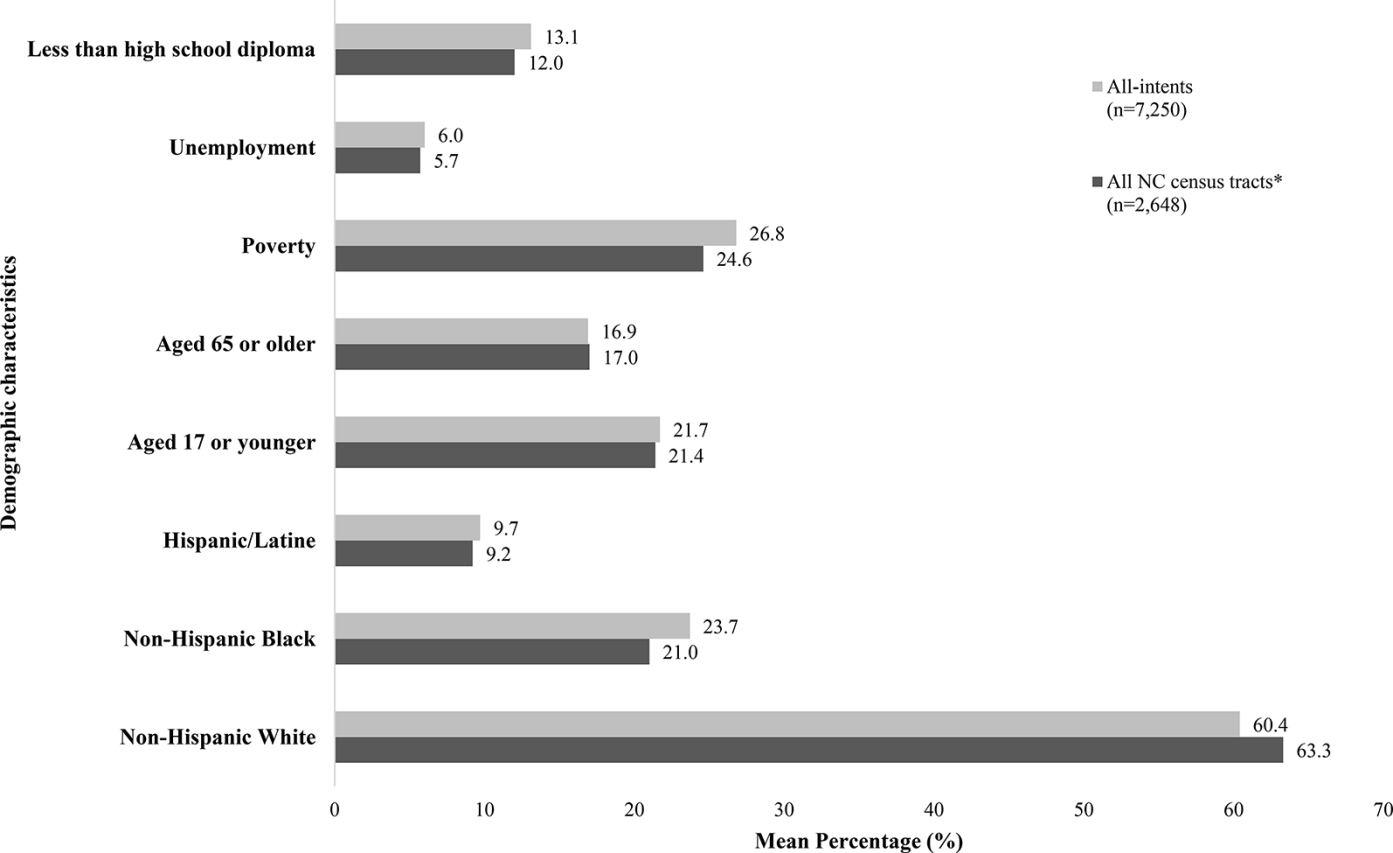
Community level characteristics of NC census tracts, including tracts with EMS encounters related to firearm injuries (all-intents), 2021–2022.* Removed 12 census tracts with 0 population

**Fig. 2:**
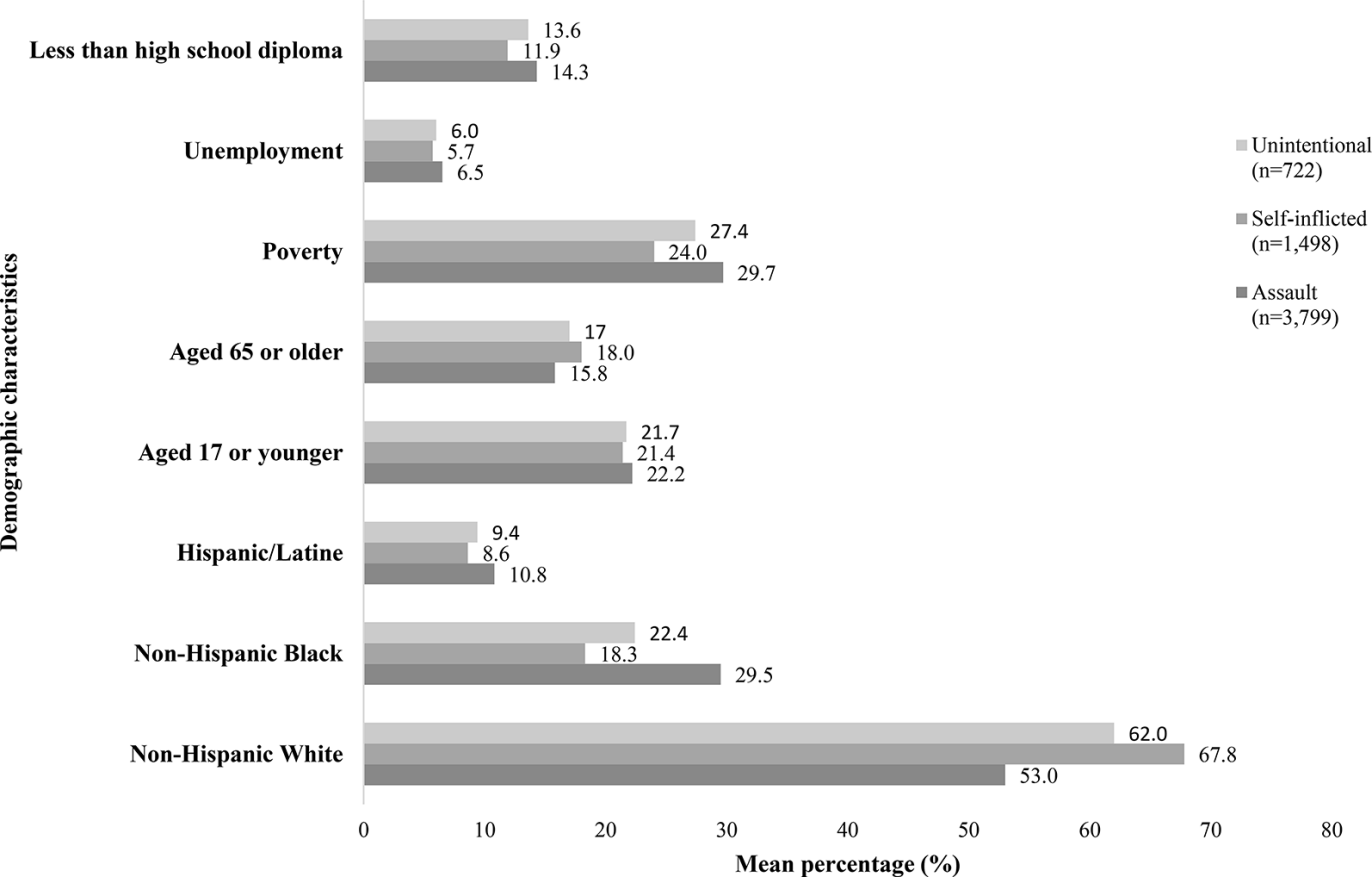
Community level characteristics of NC census tracts with EMS encounters related to firearm injuries, stratified by injury intent, 2021–2022*.  *There are 1,231 firearm injury encounters were classified as undetermined that were not included in the analysis

Figures 1 and 2 show descriptive and summary statistics for total firearm injuries and firearm injuries by intent across various demographic groups, including race, ethnicity, age, and socioeconomic status within NC’s communities (as indicated by census tract) for 2021–2022. During this period, there were 7,250 firearm injuries across all 2,648 census tracts. Assault was the leading cause of firearm injuries (*n* = 3,799), followed by self-inflicted injuries (*n* = 1,498), unintentional injuries (*n* = 722), and undetermined (*n* = 1,231). In addition, the percentage of unemployment, poverty, and individuals age 25 + with less than a high school diploma was slightly higher in census tracts with firearm injuries, as compared to all NC census tracts.

Demographic and socioeconomic characteristics differed across intent type (Fig. 2). Communities with assault-related firearm injures had a higher percentage of NH-White (53.0%), followed by NH-Black (29.5%) and then Hispanic (10.8%). The percentage of NH-Black population was higher in communities impacted by assault-related firearm injuries when compared to the total NC population. In communities impacted by intentional self-inflicted firearm injuries; the majority of the population were identified as NH-White (67.8%) when compared to the total NC population (63.3%).

The bivariate associations between SVI and firearm injury rates across different firearm intents in NC are shown in Additional file 1: Table S1, and S2. In 2021–2022, the incidence of all-intent firearm injury was 68.39 per 100,000 population in NC. The rate of all-intent firearm injury in high-SVI communities (136.58 per 100,000 population) was approximately 4.7 times greater than the rate in low-SVI communities (28.87 per 100,000 population). Furthermore, the assault firearm injury rate was 10.2 times higher in high-SVI communities (100.03 per 100,000 population) than in low-SVI communities (9.79 per 100,000 population). Rates of self-inflicted and unintentional firearm injuries were similar in the high-SVI and low-SVI communities. When removing race/ethnic minority status from the overall SVI (Additional file 1: Tables S3, S4, S5 and S6), we observed a positive relationship between high-SVI communities and all-intent firearm injuries (IRR: 5.01, 95% CI: 4.46–5.63), assaults (IRR: 9.81, 95% CI: 8.20-11.74), and unintentional firearm injuries (IRR: 2.44, 95% CI: 1.88–3.17), as compared to low-SVI communities. However, there was no significant association between high SVI and self-inflicted firearm injuries (IRR: 0.99, 95% CI: 0.83–1.18). Additionally, the rate of all-intent and assault firearm injuries increased with each increase in SVI quartile, while this was not the case for intentional self-inflicted and unintentional firearm injuries.

**Fig. 3:**
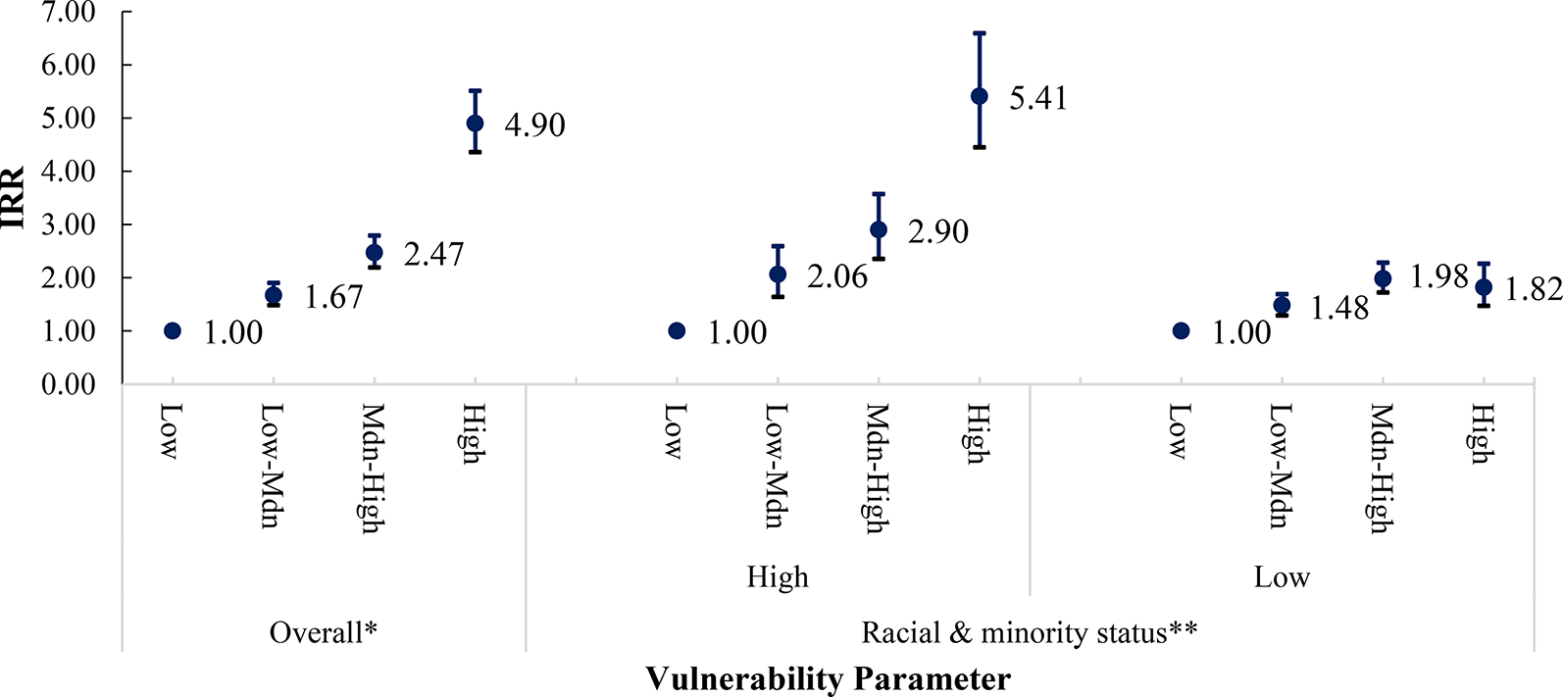
Bivariate association between SVI and rates of all-intents firearm injury. *The SVI was recalculated with racial and ethnic minority status (Theme 3) removed and categorized as 4 groups: low, low-medium, medium-high, and high, based on the CDC/ATSDR definition.  ** Median percentage percentile of census tract population falling into a racial or ethnic minority group: 50th percentile

**Fig. 4:**
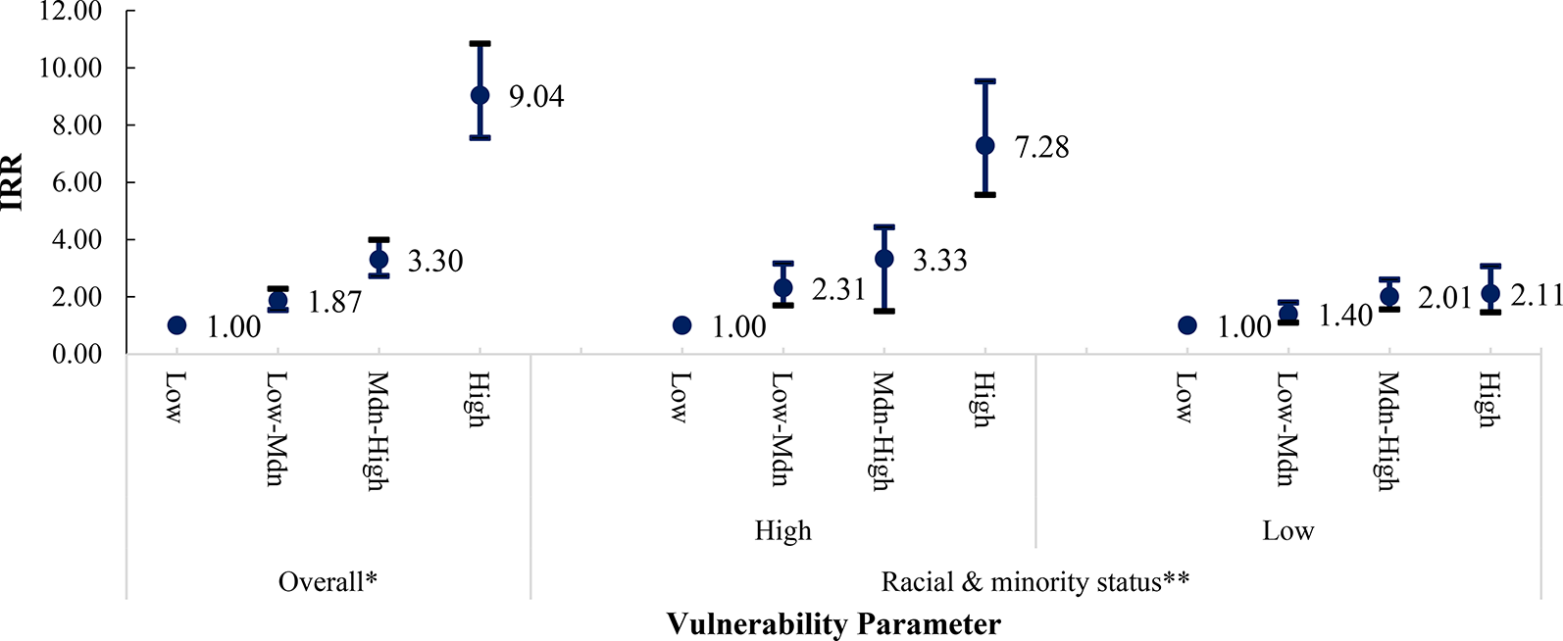
Bivariate association between SVI and rates of assault firearm injury. *The SVI was recalculated with racial and ethnic minority status (Theme 3) removed and categorized as 4 groups: low, low-medium, medium-high, and high, based on the CDC/ATSDR definition. ** Median percentage percentile of census tract population falling into a racial or ethnic minority group: 50th percentile

**Fig. 5:**
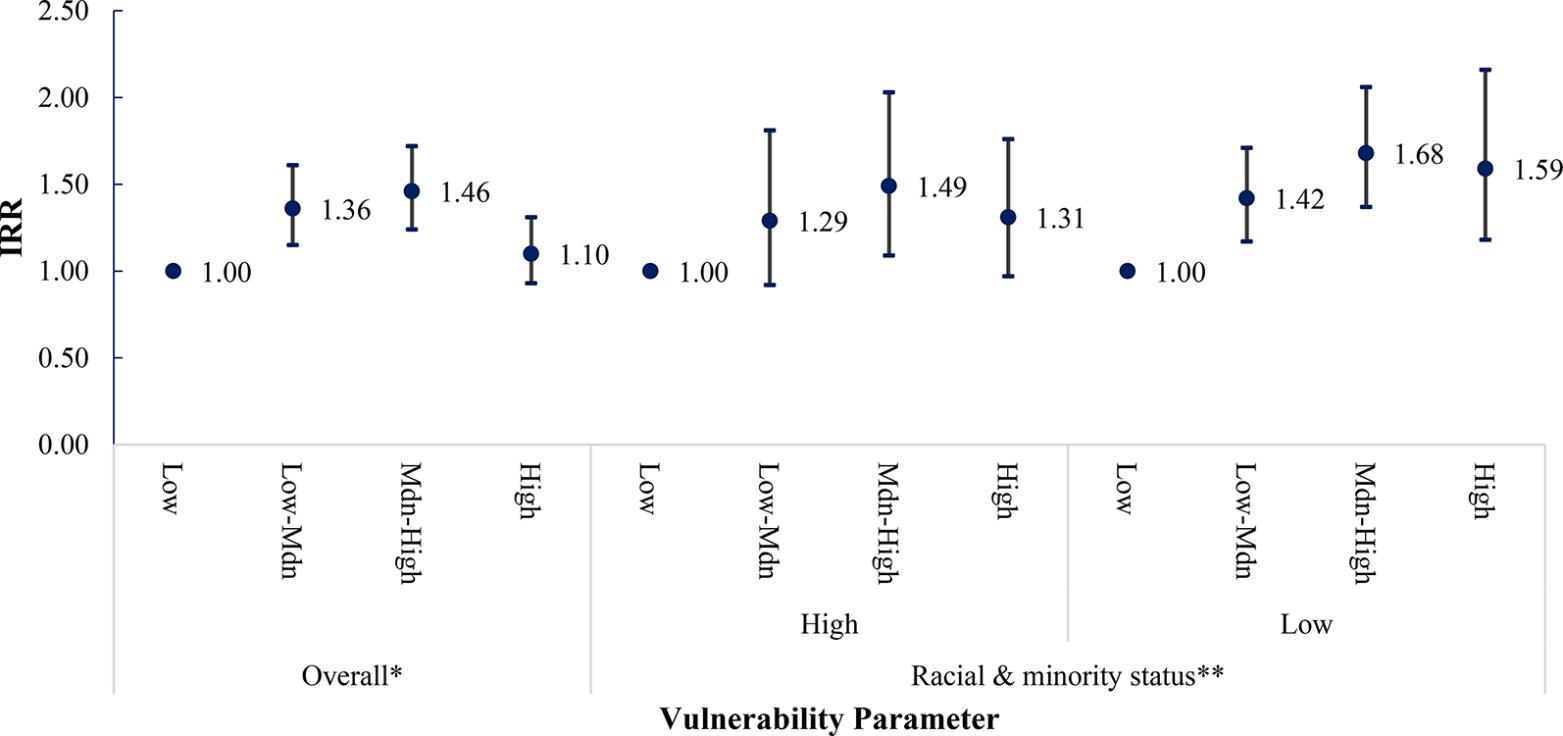
Bivariate association between SVI and rates of self-inflicted firearm injury. *The SVI was recalculated with racial and ethnic minority status (Theme 3) removed and categorized as 4 groups: low, low-medium, medium-high, and high, based on the CDC/ATSDR definition ** Median percentage percentile of census tract population falling into a racial or ethnic minority group: 50th percentile

**Fig. 6:**
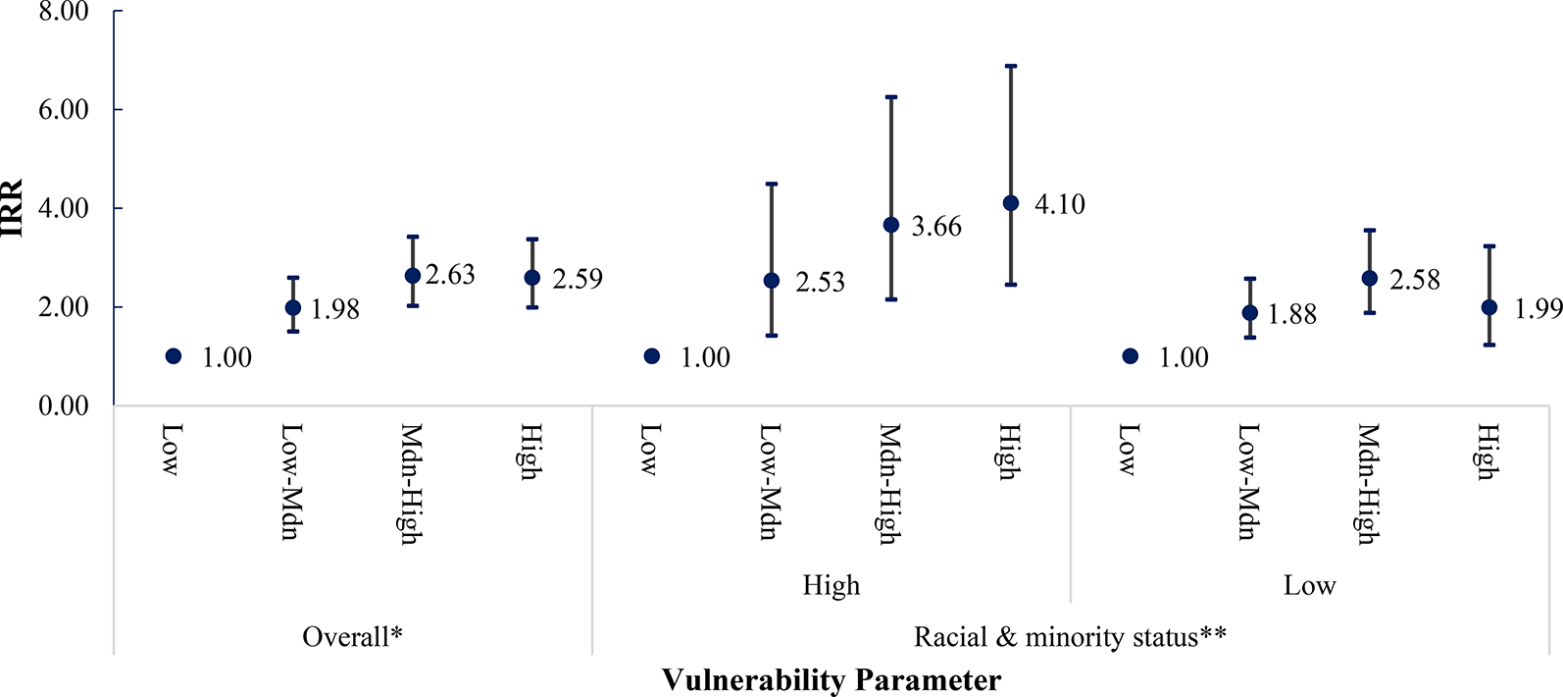
Bivariate association between SVI and rates of unintentional firearm injury.*The SVI was recalculated with racial and ethnic minority status (Theme 3) removed and categorized as 4 groups: low, low-medium, medium-high, and high, based on the CDC/ATSDR definition ** Median percentage percentile of census tract population falling into a racial or ethnic minority group: 50th percentile

We observed evidence of modification by race and ethnic composition on the relationship between SVI and firearm injury incidence at the community level, with the strength of modification observed differing by firearm injury intent (Figs. 3, 4, 5 and 6). Among census tracts with a high racial/ethnic minority population, the rate of all-intent firearm injury in the high-SVI quartile was 5.41 times (95% CI: 4.45–6.59) that of the low SVI quartile. Whereas among census tracts with a low racial/ethnic minority population, the rate of all intent firearm injuries in the high-SVI quartile was only 1.82 times (95% CI: 1.47–2.26) that of the low-SVI quartile.

Similar modification was observed for assault-related firearm injuries. Among census tracts with a high racial/ethnic minority population, the rate of firearm injuries due to assault in the high-SVI quartile was 7.28 times (95% CI: 5.56–9.52) that in the low SVI quartile. However, the rate of firearm injuries due to assault within the high SVI quartile was only 2.21 times (95% CI: 1.46–3.07) that in the low-SVI quartile among communities with a lower percentage of individuals from a racial/ethnic minority background. Racial/ethnic minority composition also modified the relationship between SVI and unintentional firearm injury incidence, but to a lesser extent than for assault. Interestingly, while we did not observe a positive relationship between SVI and self-inflicted firearm injuries among communities with a high racial/ethnicity minority composition, we did find differences among communities with lower racial/ethnic minority populations. High-SVI communities with a lower population of racial/ethnic minorities had an intentional self-inflicted firearm injury rate 1.57 times that of census tracts identified as low SVI.

## Discussion

We analyzed the relationship between overall firearm injury incidents, based on NC EMS encounters, and social vulnerability, as well as stratum-specific estimates for assault, intentional-self-inflicted, and unintentional firearm injury incidents, at the community (indicated as census tract) -level in NC. While several studies have examined this relationship (Polcari et al. 2023;  Kwon et al. 2023), to our knowledge, this is the first study to use population-based EMS encounter data to examine the relationship between firearm violence and SVI at a statewide level.

We observed a positive relationship between higher –community-level SVI and rates of all-intent, assaults, and unintentional firearm injuries, though results were inconsistent for intentional self-inflicted injuries. When we removed race/ethnic minority status and stratified by census tracts with a high (above the 50th percentile) versus a low racial/ethnic minority composition (at or below the 50th percentile) this positive association persisted. However, estimates were not consistent across all strata. Therefore, our study supports that race/ethnic minority status at the community-level is a modifier of the relationship between SVI and firearm injury incidence between areas with higher proportions of racial and ethnic minority populations versus areas with equal or lower racial and ethnic minority populations. Additionally, the extent of the modification observed varied by firearm injury intent.

Our findings suggest that SVI is a critical factor in understanding and addressing the unique vulnerabilities and risk factors communities face regarding firearm violence. Underlying public health disparities, rooted in processes and practices of systemic racism, exist in these communities beyond what is captured by SVI, which can lead to increased burden of firearm violence (Poulson et al. 2021;  Mehranbod et al. 2022). For example, residential segregation and discrimination have lasting impacts on economic opportunities and wealth accumulation within the US, leading to racial differences in economic, housing, and transportation resources (Snyder et al. 2024). While these disparities are all measured as indicators of social vulnerability and influence firearm injury and death overall as well as across communities, SVI does not fully capture the policies and practices that create and maintain lasting racial and economic disparities in firearm outcomes (Mehranbod et al. 2022; Betz et al. 2021).

To our knowledge, no other study has removed the minority status component from the SVI for stratification analysis, therefore we were unable to ascertain how this impacts the validity of SVI. In our study, we did not find substantial differences in overall SVI with or without the minority status component theme. Several studies have documented persistent racial and ethnic disparities for firearm injuries and continue to highlight the need for research that explores SVI within racial and ethnic groups to better develop effective solutions (Poulson et al. 2021;  Mehranbod et al. 2022).

Although this is the first study to use geocoded EMS data to examine the relationship between firearm injury incidents and SVI in NC, the study has several limitations. First, a significant limitation is the assumption that the 2020 SVI data remained stable through 2021 and 2022. The COVID-19 pandemic brought about massive sociological shifts that could have altered community-level social vulnerability. Any changes in SVI during these years could influence the observed associations and should be considered when interpreting the results. Second, the potential inconsistency in documentation of firearm injuries in EMS among emergency responders with varying degrees of training and differences in treatment and transport policies and protocols, potentially resulting in an underestimation of the number of EMS encounters identified as related to firearm violence. Third, although firearm injury intent was manually assigned through a record-level review of the assigned injury mechanism codes and EMS narratives, the resulting intent coding may still be inaccurate if the reporting of intent to or by EMS was inaccurate or incomplete. Next, since this study estimated the association between SVI and firearm injury at the community-level, it is susceptible to the ecological fallacy; therefore, it is not appropriate to make individual-level inferences based on the results of our study. Additionally, combining all racial and ethnic groups in the analysis may underscore important differences, as different groups experience varying levels of burden from SVI and firearm violence; future research should disaggregate these groups to better understand the association between race/ethnicity, SVI, and firearm violence. Lastly, since we examined the effects of community-level attributes aggregated at the census tract level, our study may be affected by two fundamental methodological problems, modifiable areal unit problem (MAUP) and uncertain geographic context problem (UGCoP) (Kwan and Kwan 2012). However, census tract-level data is the smallest geographic scale available for SVI and serves as the best proxy for community factors.

Despite limitations, this study has important public health implications. Our study showed significant disparities in the rates of firearm injuries among NC communities, with higher rates observed in communities with high SVI. In addition, we found that the effects of SVI were more pronounced in racial/ethnic minority communities, demonstrating the urgent need to address underlying social determinants of health, such as access to education, employment, and social welfare programs. Due to complexities and inequities associated with and contributing to community firearm injury, systemic interventions are needed to address the situation. For example, income support policies (Rowhani-Rahbar et al. 2022), social and public health services (Kim et al. 2019), the Earned Income Tax Credit (Lenhart and Lenhart 2021), and reducing income inequality (Johnson et al. 2021), have led to reductions in firearm injuries. Moreover, the Cardiff Violence Prevention Model, which combines and maps anonymous hospital and law enforcement data to help create and evaluate local place-based solutions, has been shown to reduce violence and injuries (Stiles et al. 2022;  Centers for Disease Control and Prevention 2021).

## Conclusion

This study used statewide, population-based EMS encounter data to suggest a positive association between high-SVI communities and firearm injuries compared to low-SVI communities, and racial/ethnic minority status acts as an effect measure modifier between the relationship of firearm injury incidence and SVI. Additionally, this study also identified significant disparities in the occurrence of firearm injuries among NC communities with greater proportions of racial/ethnic minorities. Multilevel contributions, such as addressing the underlying social determinants of health (e.g., education level) and community-level intervention programs, are needed to prevent firearm injuries.

## Electronic supplementary material

Supplementary Material 1

## Data Availability

No datasets were generated or analysed during the current study.

## Abbreviations

ATSDR: Agency for Toxic Substances and Disease Registry
ACS: American Community Survey
CDC: Centers for Disease Control and Prevention
EMS: Emergency medical services
IQR: Interquartile ranges
MAUP: Modifiable areal unit problem
NH: Non-Hispanic
NC: North Carolina
NC DETECT: North Carolina Disease Event Tracking and Epidemiologic Collection Tool
SVI: Social Vulnerability Index
SD: Standard deviations
UGCoP: Uncertain geographic context problem
US: United States
