# Supplementary material for: Using EMS data to explore community-level factors associated with firearm violence in North Carolina
Source: Inj Epidemiol. 2024 Oct 25;11:58. doi: 10.1186/s40621-024-00539-9 (PMC11515109; doi:10.1186/s40621-024-00539-9)
Supplement: Supplementary file 1 — Supplementary Material 1 [file 40621_2024_539_MOESM1_ESM.docx]

**Appendix Table 1. Community level characteristics and firearm injuries by intent in North Carolina (NC), 2021-2022***

| ​**Community level characteristics** | **All NC Census Tracts** (N=2,648)** | | **Census tracts with firearm injuries (n=7,250)** | | **Firearm injuries by intent***** | | | | | |
| --- | --- | --- | --- | --- | --- | --- | --- | --- | --- | --- |
|  |  |  |  |  | **Assault (n=3,799)** | | **Self-inflicted (n=1,506)** | | **Unintentional (n=722)** | |
|  | Median | IQR | Median | IQR | Median | IQR | Median | IQR | Median | IQR |
| Non-Hispanic White, % | 69.1 | 38.1 | 65.3 | 41.4 | 55.4 | 43.5 | 74 | 31.5 | 66.8 | 41.0 |
| Non-Hispanic Black, % | 14.1 | 27.9 | 17.6 | 30.8 | 25.8 | 33.7 | 11.9 | 24.0 | 16.4 | 31.0 |
| Hispanic/Latine, % | 6.4 | 9.9 | 6.8 | 10.7 | 7.9 | 11.5 | 6.2 | 9.1 | 6.5 | 10.5 |
| Aged 17 or younger, % | 21.5 | 8.3 | 21.6 | 8.0 | 22.3 | 8.2 | 21.4 | 7.3 | 21.5 | 7.6 |
| Aged 65 or older, % | 16.2 | 10.0 | 16.2 | 9.4 | 15.2 | 9.4 | 17.0 | 9.2 | 16.6 | 9.3 |
| Poverty, % | 22.9 | 19.8 | 25.2 | 18.9 | 28.2 | 20.5 | 22.5 | 16.7 | 26.4 | 16.8 |
| Unemployment, % | 4.7 | 5.0 | 5.1 | 5.3 | 5.6 | 5.5 | 4.7 | 5.0 | 5.3 | 5.4 |
| Less than high school diploma, % | 10.8 | 11.6 | 12.2 | 11.3 | 13.4 | 11.9 | 10.9 | 9.8 | 12.9 | 10.4 |

Abbreviations: IQR=Interquartile Range

* Community level was indicated as census tract level.

** Removed 12 census tracts with 0 population.

*** 1,231 firearm injury encounters were classified as undetermined and were not included in the analysis.

| **Appendix Table 2. Association between firearm injury incidence and social vulnerability metrics, stratified by intent; North Carolina, 2021-2022*** | | | | | | | | | | | | |
| --- | --- | --- | --- | --- | --- | --- | --- | --- | --- | --- | --- | --- |
| ​**Vulnerability Parameter** | **All NC Census Tracts**** | | | **Intentional assault** | | | **Intentional self-inflicted** | | | **Unintentional** | | |
|  | **Rate** | **IRR** | **95% CI** | **Rate** | **IRR** | **95% CI** | **Rate** | **IRR** | **95% CI** | **Rate** | **IRR** | **95% CI** |
| Overall SVI*** |  |  |  |  |  |  |  |  |  |  |  |  |
| Low | 28.87 | Ref | Ref | 9.79 | Ref | Ref | 12.24 | Ref | Ref | 3.58 | Ref | Ref |
| Low-Medium | 46.57 | 1.62 | 1.43, 1.85 | 17.42 | 1.80 | 1.48, 2.20 | 16.17 | 1.32 | 1.12, 1.56 | 6.77 | 1.89 | 1.45, 2.48 |
| Medium-High | 68.61 | 2.38 | 2.11, 2.68 | 31.89 | 3.30 | 2.73, 3.99 | 16.97 | 1.38 | 1.17, 1.63 | 8.74 | 2.44 | 1.88, 3.17 |
| High | 136.58 | 5.01 | 4.46, 5.63 | 100.03 | 9.81 | 8.20, 11.74 | 12.27 | 0.99 | 0.83, 1.18 | 8.76 | 2.44 | 1.88, 3.17 |
| Abbreviation: SVI: Social Vulnerability Index  * Rate was expressed per 100,000 population  ** Removed 12 census tracts with 0 population.  *** The SVI categorization was categorized as 4 groups as low, low-medium, medium-high, and high based on the CDC/ATSDR definition. | | | | | | | | | | | | |

| **Appendix Table 3. Bivariate association between SVI and rates of all-intents firearm injury, North Carolina, 2021-2022*** | | | | |
| --- | --- | --- | --- | --- |
| **Vulnerability Parameter** | **Overall** | | **Racial & Ethnic Minority Status** | |
|  |  |  | **Above the median** | **Less or equal to the median** |
|  | **Rate** | **IRR (95% CI)** | **IRR (95% CI)** | **IRR (95% CI)** |
| Overall SVI** |  |  |  |  |
| Low | 28.87 | Ref | Ref | Ref |
| Low-Medium | 46.57 | 1.62 (1.43, 1.85) | 2.06 (1.64, 2.59) | 1.48 (1.29, 1.69) |
| Medium-High | 68.61 | 2.38 (2.11, 2.68) | 2.90 (2.35, 3.57) | 1.98 (1.72, 2.28) |
| High | 136.58 | 5.01 (4.46, 5.63) | 5.41 (4.45, 6.59) | 1.82 (1.47, 2.26) |

Abbreviations: SVI= Social Vulnerability Index; IRR= Incidence Rate Ratio; CI= Confidence Interval

* Rate was expressed per 100,000 population

** The SVI categorization was categorized as 4 groups as low, low-medium, medium-high, and high based on the CDC/ATSDR definition.

| **Appendix Table 4. Bivariate association between SVI and rates of intentional assault firearm injury, North Carolina, 2021-2022*** | | | | |
| --- | --- | --- | --- | --- |
| **Vulnerability Parameter** | **Overall** | | **Racial & Ethnic Minority Status** | |
|  |  |  | **Above the median** | **Less or equal to the median** |
|  | **Rate** | **IRR (95% CI)** | **IRR (95% CI)** | **IRR (95% CI)** |
| Overall SVI** |  |  |  |  |
| Low | 9.79 | Ref | Ref | Ref |
| Low-Medium | 17.42 | 1.80 (1.48, 2.20) | 2.31 (1.70, 3.16) | 1.40 (1.10, 1.80) |
| Medium-High | 31.89 | 3.30 (2.73, 3.99) | 3.33 (2.50, 4.43) | 2.01 (1.56, 2.60) |
| High | 100.03 | 9.81 (8.20, 11.74) | 7.28 (5.56, 9.53) | 2.11 (1.46, 3.07) |

Abbreviations: SVI= Social Vulnerability Index; IRR= Incidence Rate Ratio; CI= Confidence Interval

* Rate was expressed per 100,000 population

** The SVI categorization was categorized as 4 groups as low, low-medium, medium-high, and high based on the CDC/ATSDR definition.

| **Appendix Table 5. Bivariate association between SVI and rates of intentional self-inflicted firearm injury, North Carolina, 2021-2022*** | | | | |
| --- | --- | --- | --- | --- |
| **Vulnerability Parameter** | **Overall** | | **Racial & Ethnic Minority Status** | |
|  |  |  | **Above the median** | **Less or equal to the median** |
|  | **Rate** | **IRR (95% CI)** | **IRR (95% CI)** | **IRR (95% CI)** |
| Overall SVI** |  |  |  |  |
| Low | 12.24 | Ref | Ref | Ref |
| Low-Medium | 16.17 | 1.32 (1.12, 1.56) | 1.31 (0.93, 1.84) | 1.41 (1.17, 1.71) |
| Medium-High | 16.97 | 1.38 (1.17, 1.63) | 1.51 (1.11, 2.07) | 1.68 (1.37, 2.05) |
| High | 12.27 | 0.99 (0.83, 1.18) | 1.31 (0.97, 1.76) | 1.57 (1.16, 2.12) |

Abbreviations: SVI= Social Vulnerability Index; IRR= Incidence Rate Ratio; CI= Confidence Interval

* Rate was expressed per 100,000 population

** The SVI categorization was categorized as 4 groups as low, low-medium, medium-high, and high based on the CDC/ATSDR definition.

| **Appendix Table 6. Bivariate association between SVI and rates of unintentional firearm injury, North Carolina, 2021-2022*** | | | | |
| --- | --- | --- | --- | --- |
| **Vulnerability Parameter** | **Overall** | | **Racial & Ethnic Minority Status** | |
|  |  |  | **Above the median** | **Less or equal to the median** |
|  | **Rate** | **IRR (95% CI)** | **IRR (95% CI)** | **IRR (95% CI)** |
| Overall SVI* |  |  |  |  |
| Low | 3.58 | Ref | Ref | Ref |
| Low-Medium | 6.77 | 1.89 (1.45, 2.48) | 2.53 (1.42, 4.49) | 1.88 (1.38, 2.57) |
| Medium-High | 8.74 | 2.44 (1.88, 3.17) | 3.66 (2.15, 6.25) | 2.58 (1.88, 3.55) |
| High | 8.76 | 2.44 (1.88, 3.17) | 4.10 (2.45, 6.88) | 1.99 (1.23, 3.23) |

Abbreviations: SVI= Social Vulnerability Index; IRR= Incidence Rate Ratio; CI= Confidence Interval

* Rate was expressed per 100,000 population

** The SVI categorization was categorized as 4 groups as low, low-medium, medium-high, and high based on the CDC/ATSDR definition
